# The metabolic activity of denitrifying microorganisms accumulating polyphosphate in response to addition of fusel oil

**DOI:** 10.1007/s00449-018-2022-0

**Published:** 2018-10-05

**Authors:** Agnieszka Tuszynska, Magdalena Kaszubowska, Przemyslaw Kowal, Slawomir Ciesielski, Jacek Makinia

**Affiliations:** 1 Department of Sanitary Engineering, Faculty of Civil and Environmental Engineering, Gdansk University of Technology, Narutowicza Street 11/12, 80-233 Gdansk, Poland; 2 Department of Environmental Sciences, Faculty of Environmental Sciences, University of Warmia and Mazury in Olsztyn, Sloneczna 45G, 10-917 Olsztyn, Poland

**Keywords:** Alternative carbon source, Fusel oil, Activated sludge, Anoxic N and P removal

## Abstract

The effect of distillery waste product (fusel oil) as an alternative external organic carbon source (EOCS) was investigated in terms of the metabolic properties of denitrifying polyphosphate accumulating organisms (DPAOs). Samples of the non-acclimated biomass were collected from a local full-scale wastewater treatment plant employing A^2^/O type bioreactors. The acclimated biomass was obtained after cultivation (with fusel oil added) in a bench-scale reactor with a process configuration similar to the full-scale bioreactor. Changes in the functional properties of the biomass were investigated by measuring the phosphate release/uptake rates (PRRs and PURs), and nitrate utilization rates (NURs) with fusel oil in anaerobic-anoxic batch tests. Furthermore, a validated extended Activated Sludge Model no 2d (ASM2d) was used as a supporting tool to analyze the experimental results and estimate the contribution of DPAOs to the overall denitrification. In the non-acclimated biomass with fusel oil, the PRRs, PURs and NURs were low and close to the rates obtained in a reference test without adding EOCS. With the acclimated biomass, the PUR and NUR increased significantly, i.e., 3.5 and 2.7 times, respectively. In the non-acclimated biomass, approximately 60.0 ± 3.6% and 20.0 ± 2.2% of the total NUR was attributed to the utilization of endogenous carbon and examined EOCS, respectively. The remaining portion (20% of the total NUR) was attributed to PHA utilization (linked to PO_4_-P uptake) by DPAOs. With the acclimated biomass, the contribution of the EOCS to the NUR increased to approximately 60%, while the contribution of the endogenous carbon source decreased accordingly. Very accurate predictions of PURs and NURs (*R*^2^ = 0.97–1.00) were obtained with the extended ASM2d. Based on model simulations, it was estimated that the activity of DPAOs and denitrifying ordinary heterotrophic organisms corresponded to approximately 20% and 80% of the total NUR, respectively.

**Electronic supplementary material:**

The online version of this article (10.1007/s00449-018-2022-0) contains supplementary material, which is available to authorized users.

## Introduction

The successful operation of existing biological nutrient removal (BNR) wastewater treatment plants (WWTPs) depends to a great extent on the availability of sufficient amounts of appropriate (biodegradable) organic compounds (carbon sources). In the combined N and P systems, these compounds should satisfy the needs of three microbial groups, including denitrifying “ordinary” heterotrophs, “ordinary” polyphosphate accumulating organisms (PAOs)—performing enhanced biological P removal (EBPR) in the traditional anaerobic/aerobic cycle—and denitrifying PAOs (DPAOs)—performing EBPR and denitrification in the provision of the anaerobically stored poly-hydroxy-alkanoates (PHA). The combination of the two processes by DPAOs is a sustainable solution as it can save aeration and carbon source required for denitrification, resulting in the reduction of energy consumption and operational costs [1–4].

Due to the limited amounts of biodegradable organic compounds often encountered in the influent wastewater, the three groups of microorganisms have to compete for the same substrate. To overcome the limitation for denitrification, either “conventional” or “alternative” external organic carbon sources (EOCS). The first group includes commercial products, such as methanol, ethanol, acetic acid, sodium acetate or glucose. Despite their proved efficiency, several recent studies emphasized economical barriers for the practical use in WWTPs [5–8]. Due to high costs of the commercial compounds, various industrial byproducts or waste materials have received more attention as the “alternative” EOCS. Moreover, such an approach is in accordance with the principle of “zero waste” strategy implemented in the European Union to stimulate the development of new waste reuse technologies [9].

Results of numerous studies e.g., [10–18] have indeed confirmed that the characteristics of various branches of the agro-food industry are beneficial for enhancing denitrification in WWTPs in terms of the process rate and efficiency. Gu and Onnis-Hayden (2010) [19] presented a practical evaluation protocol of the alternative EOCS for denitrification. The protocol, recommended by the Water Environment Research Foundation (WERF) for the WWTP operators, provides a guidance to evaluate the feasibility of using specific EOCS in full-scale WWTPs.

However, one of the most important deficiencies of that protocol is that the document does not consider the effect of dosing EOCS on interactions between denitrification and EBPR occurring in the combined N and P systems. The aim of this study was to investigate changes in the metabolic activity of the DPAOs under anaerobic/anoxic conditions in response to addition of a distillery waste product (fusel oil). This compound is potentially a very favorable carbon source for denitrification due to the advantageous composition, i.e., high chemical oxygen demand (COD) concentrations and high COD/N ratios, which has been confirmed in a few earlier studies [20, 21]. However, the anoxic interactions between denitrification and EBPR in the presence of fusel oil have not been reported so far. Furthermore, a complex activated sludge model was used as a supporting tool to analyze the experimental results and estimate the contribution of DPAOs to the overall denitrification under different environmental conditions. The investigations were extended with a microbial analysis using the 16S rRNA PCR-DGGE (Polymerase Chain Reaction-Denaturing Gradient Gel Electrophoresis) technique. To characterize the dominant heterotrophic denitrifying bacteria and DPAOs in the acclimated biomass, clone libraries of *nirS* and *nirK* genes were prepared and analyzed with bioinformatics tools.

## Methodology

### Origin of the biomass

#### Non-acclimated biomass

The activated sludge samples were collected from a large municipal WWTP “Wschod” (600,000 PE) in the city of Gdansk (northern Poland). A modified A^2^/O process configuration is currently employed for BNR in the biological stage. The WWTP meets the European Union effluent criteria for large WWTPs, i.e., effluent concentrations of total *N* (TN) = 10 mgN·L^−1^ and total *P* (TP) = 1 mgP·L^−1^.

#### Acclimation of biomass to fusel oil

The acclimation of processed biomass to fusel oil was conducted in a bench-scale continuous flow reactor with the process configuration similar to the full-scale A^2^/O bioreactor. The total working volume of the reactor was 30.0 L with the volumetric ratios of 4:9:14 L of the subsequent compartments (anaerobic : anoxic : aerobic). The reactor was fed with the primary effluent from the “Wschod” WWTP and the wastewater inflow rate to the reactor was kept constant at 1.13 L·h^−1^ to obtain a similar hydraulic retention time (HRT) to the average HRT in the full-scale bioreactor. Fusel oil with the total COD concentration of 1,690,000 mgO_2_·L^−1^ was added to the anoxic compartment in the total amount of 1.5 mL·day^−1^. The metered amount of fusel oil was mixed with water in proportion of 1:30 and dispensed periodically using a peristatic pump Heidolph PD 5001 (Schwabach, Germany). During the acclimation period, the return activated sludge and mixed liquor recirculations were, respectively, set to 150% and 500% of the influent flow rate. The solids retention time (SRT) was controlled at a constant level of 20 days based on the mixed liquor suspended solids (MLSS) concentrations in the reactor. Those concentrations varied in the range of approximately 3000–3300 mg·L^−1^. The set point for dissolved oxygen concentration in the aerobic compartment was 2.0 mgO_2_·L^−1^. In total, the system was operated for over 100 days, but the acclimation period of approximately of 50 days was needed to achieve the maximum NURs. More information about the system performance can be found elsewhere [22]. For the batch experiments with the acclimated biomass, described in the present study, the processed biomass was used after 53 days of acclimation.

### External organic carbon sources

The EOCSs examined in the batch experiments comprised the principal substrate for PAOs (acetate) and a fermentation byproduct from distilleries (fusel oil).

The basic characteristics of acetate and fusel oil used in the present study (10 samples from 7 different distilleries) are outlined in Table 1. The composition of the fusel oil revealed a high content of organic compounds (COD) and high value of the COD/TN ratio (approximately 1800). Moreover, the detailed composition of fusel oil was investigated with the gas chromatography with respect to the content of selected 29 organic compounds.

**Table 1:** Basic characteristics of the different electron donors used in the lab-scale experiments

Parameter	COD (mgO_2_·L^−1^)	TOC (mgC·L^−1^)	TN (mgN·L^−1^)	NH_4_-N (mgN·L^−1^)	TP (mgP·L^−1^)
Fusel oil	1,690,000–1,890,000	412,000–415,000	960–1040	1.9–2.5	0.2
Acetate	913,000	–	–	–	–

### Experimental procedure

A series of two-phase anaerobic-anoxic batch experiments were carried out in plexiglass batch reactors (the working volume of 4.0 L), which were equipped with electrodes and probes (WTW, Germany) for a continuous monitoring of pH (SenTix 21) and temperature (CellOx 325). The temperature set point was 20.0 ± 0.5 °C, ensured by a circulation pump with a controlled cooling water valve. The pH was controlled at 7.0 ± 0.5 which is optimal for the dominance of PAOs [23]. Each experiment lasted 7.5 h, including 2.5 h of the anaerobic phase followed by 5.0 h of the anoxic phase.

The batch experiments were carried out with non-acclimated (Tests 1–5) and acclimated to fusel oil-biomass (Test 6 and 7). Apart from the reference test (Test 1, performed without any EOCS), different combinations of the examined EOCS were dosed at the beginning of both anaerobic and anoxic phase as shown in Table 2. The dosage of acetate (A) and fusel oil (FO) was 0.46 mL and 0.16–0.25 mL, respectively. The dosages of EOCS increased COD concentrations in the reactors in the range of approximately 120 to 200 mgO_2_·L^−1^.

**Table 2:** Examined EOCS and their dosages (mL) in the two-phase anaerobic-anoxic batch experiment

	Test 1 RT (na)	Test 2 A-FO (na)	Test 3 FO-FO (na)	Test 4 A-FO/PIX (na)	Test 5 FO-FO/PIX (na)	Test 6 FO-FO (a)	Test 7 FO-FO/PIX (a)
Biomass	Non-acclimated biomass (na)					Acclimated biomass (a)	
The dosages of EOCS (mL)							
Anaerobic phase^a^	Reference test (RT) (mixed liquor of wastewater and activated sludge) without EOCS	Acetate (0.46)	Fusel oil (0.16–0.18)	Acetate (0.46)	Fusel oil (0.16–0.18)	Fusel oil (0.25)	Fusel oil (0.25)
Anoxic phase^b^		Fusel oil (0.25)	Fusel oil (0.16–0.22)	Fusel oil (0.25) PIX 113 (2.50)	Fusel oil (0.16–0.18) PIX 113 (2.50)	Fusel oil (0.25)	Fusel oil (0.25) PIX 113 (2.50)

^a^The dosage of EOCS at the beginning of the anaerobic phase

^b^The dosage of EOCS at the beginning of the anoxic phase

In each test, at the beginning of the anoxic phase, potassium nitrate (KNO_3_) was added in the amount of 525 mg (for the non-acclimated biomass) and 1315 mg (for the acclimated biomass) to increase the nitrate concentration in the reactor by approximately 19 ± 1 and 48 ± 2 mgN.L^−1^, respectively. In the latter case, the higher concentrations applied resulted from much higher denitrification capabilities of the acclimated biomass. Consequently, the COD dosages had to be adjusted to obtain the initial COD: *N* ratios > 4 to avoid the limitation of the denitrification process. This resulted in the range of 120–200 mg COD.L^−1^ of the initial COD in the batch experiments. Therefore, the doses of fusel oil varied from 0.16 to 0.25 mL. In both cases, the NO_3_-N concentrations were sufficient to promote the optimal anoxic PO_4_-P uptake activity. The characteristics of the initial conditions—mixed liquor of wastewater and activated sludge—in the two-phase anaerobic-anoxic batch experiments are presented in Table 3.

**Table 3:** Characteristics of the initial conditions (mixed liquor of wastewater and activated sludge) in the two-phase anaerobic-anoxic batch experiments (average concentrations ± standard deviations, *n* = 3–4 repetitions for each experiment)

Parameter	Test 1 RT (na)	Test 2 A-FO (na)	Test 3 FO-FO (na)	Test 4 A-FO/PIX (na)	Test 5 FO-FO/PIX (na)	Test 6 FO-FO (a)	Test 7 FO-FO/PIX (a)
Biomass	Non-acclimated biomass (n-ab)					Acclimated biomass (ab)	
COD, (mgO_2_.L^−1^)	47.0 ± 3.0	35.0 ± 3.0	35.0 ± 2.0	37.0 ± 4.0	38.0 ± 3.0	40.0 ± 5.0	41.0 ± 4.0
TP (mgP.L^−1^)	9.1 ± 1.3	7.5 ± 0.9	6.9 ± 1.1	9.4 ± 1.5	8.3 ± 1.4	4.2 ± 0.8	2.5 ± 0.5
NO_3_-N (mgN.L^−1^)	0.3 ± 0.1	0.2 ± 0.1	0.4 ± 0.1	0.3 ± 0.1	0.30 ± 0.1	0.5 ± 0.1	0.4 ± 0.1
NO_2_-N (mgN.L^−1^)	0.08 ± 0.03	0.02 ± 0.01	0.03 ± 0.01	0.02 ± 0.01	0.03 ± 0.01	0.01 ± 0.01	0.03 ± 0.01
MLSS (mg.L^−1^)	2829 ± 47	3117 ± 55	3010 ± 65	3335 ± 83	2929 ± 77	3128 ± 98	3208 ± 71
pH	7.0 ± 1.1	7.1 ± 1.5	7.3 ± 1.0	7.2 ± 1.5	6.9 ± 1.3	7.3 ± 0.8	7.2 ± 0.9

To evaluate the contribution of DPAOs to the increased denitrification rates and efficiency, chemical precipitation of PO_4_-P was performed in three tests (Table 2), including Tests 4 and 5 (with non-acclimated biomass) and Test 7 (with acclimated biomass). At the end of the anaerobic phase, mixing was turned off to allow for sedimentation of the activated sludge flocs. The liquid supernatant was decanted for precipitation in a separate beaker. The precipitation process was carried out using ferric chloride (III) as a commercial product PIX 113 (Kemira, Finland). The PIX with 35% of ferric chloride solution dose amounted 2.5 mL. After the precipitation, the supernatant was decanted and transferred back to the batch reactor to continue the measurements under anoxic conditions.

Altogether, seven kinds of the experiments (repeated 3–4 times) were carried out, including five and two kinds with the non-acclimated and acclimated biomass, respectively.

### Analytical methods

Before each analysis, the withdrawn samples of the mixed liquor were filtered under vacuum pressure through a 1.2 µm pore size nitrocellulose filter (Millipore, USA). Total nitrogen (TN) concentrations were determined using a TOC/TN analyzer (Shimadzu Corp., Japan). Concentrations of nitrate (NO_3_-N) and phosphate (PO_4_-P) were determined using cuvette tests in Xion 500 spectrophotometer (Dr Lange GmbH, Germany). The analytical procedures, which were adopted by Dr Lange and Shimadzu, followed the Standard Methods [24]. Total and volatile suspended solids (TSS and VSS) were measured by the gravimetric methods in accordance with the Standard Methods [24].

### Model simulations

Model simulations were carried out to estimate the contributions of DPAOs and denitrifying ordinary heterotrophic organisms (DOHOs) to nitrate utilization in each experiment. The GPS-X ver. 5.0.2 program was used as a simulation platform (Hydromantis, Canada). The model used in the present study was an extension of the Activated Sludge Model No. 2d (ASM2d) as proposed by Swinarski et al. (2012) [25]. In the extended model, fusel oil was incorporated as a new state variable (S_A,1_) termed ‘‘external readily biodegradable substrate’’. The new kind of substrate was introduced to differentiate it from the original ASM2d ‘‘fermentation products’’ (assumed to be acetate), S_A_, and to denote that S_A,1_ is not available for PAOs under anaerobic conditions, but it can be consumed by PAOs under anoxic and aerobic conditions. The rationale for introducing that new-state variable was comprehensively discussed in [22, 25]. The expanded ASM2d was implemented in GPS-X using a special utility called “Model Developer”, and subsequently calibrated and validated based on results of the comprehensive laboratory experiments and 96-h measurement campaign in the full-scale bioreactor [25]. In the present study, values of the kinetic and stoichiometric coefficients were adopted from [25] with only minor adjustments for PO_4_-P release and uptake. It should be noted that the activity of GAOs was negligible at the studied plant as no significant acetate utilization was observed after polyphosphate depletion in the PAO biomass [22]. Therefore, the GAOs’ metabolism was not incorporated in the model. In addition, in the experiments with the non-acclimated biomass (Tests 1–5), the initial concentrations of PAO (17% of MLVSS) and OHO (32% of MLVSS) were assumed based the simulation results of the full-scale bioreactor. The same values were also assumed for the experiments with the acclimated biomass (Tests 6–7) as the PAO/OHO proportions did not change substantially during the acclimation period as shown by Hu et al. [22].

### Calculations

**Fig. 1:**
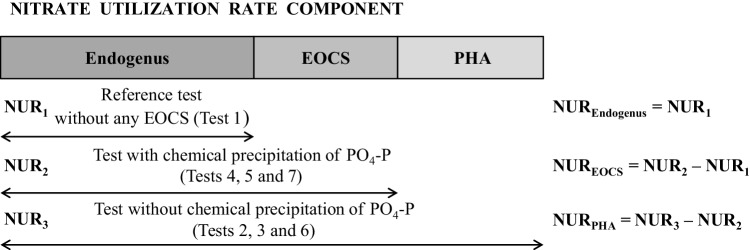
The contributions of the specific carbon sources and microbial activities on to the NURs

The phosphate release rate (PRR) (−) under anaerobic conditions and the phosphate uptake rate (PUR) (+) under anoxic conditions were calculated from the following equation:

1 $${\text{PRR or PUR }}={\text{ slope }}\left[ {{{\left( {{\text{P}}{{\text{O}}_{\text{4}}} - {\text{P}}} \right)}_{\text{i}}}} \right]\cdot{X^{ - {\text{1}}}}\cdot{t^{ - {\text{1}}}},{\text{ mgP}}\cdot{{\text{g}}_{{\text{VSS}}}}^{{ - {\text{1}}}}\cdot{{\text{h}}^{ - {\text{1}}}}$$

where, (PO_4_-P)_t_ − PO_4_-P concentration in time *t*_i_, mgP·L^−1^, X-VSS concentration, *g*_VSS_·L^−1^, *t* duration of the experiment, *h* the nitrate utilization rate under anoxic conditions was calculated from the following equation:

2 $${\text{NUR }}={\text{ }}\left[ {{{\left( {{\text{N}}{{\text{O}}_{\text{3}}} - {\text{N}}} \right)}_{\text{i}}}} \right]\cdot{{\text{X}}^{ - {\text{1}}}}\cdot{{\text{t}}^{ - {\text{1}}}},{\text{ mgN}}\cdot{{\text{g}}_{{\text{VSS}}}}^{{ - {\text{1}}}}\cdot{{\text{h}}^{ - {\text{1}}}}$$

where: (NO_3_-N)_i_ – NO_3_-N concentration in time t_i_, mgN·L^− 1^, contributions to the observed NURs (NUR_EOCS_, and NUR_PHA_, and NUR_Endogenous_) in terms of the examined carbon sources and microbial activities were calculated as presented in Fig. 1.

### Microbial analysis

#### Bacterial community structure stability

Biomass acclimation to fusel oil addition was additionally monitored by the application of the molecular microbiology tools. The 16S rDNA PCR-DGGE technique was applied to investigate shifts in the bacterial community structure after 17 and 53 days of the biomass cultivation. Activated sludge samples were collected and stored in − 20 °C prior to the analysis. Genomic DNA was isolated from the biomass by the mechanical disintegration accompanied with washing in silica columns (A & A Biotechnology, Poland) according to the method described by Ciesielski et al. (2013) [26]. The fragment of the 16S rRNA gene was amplified using F-968-GC and R-1401 primers proposed by Nübel et al. (1997) [27]. During the subsequent steps, PCR amplicons were separated in a 6% acrylamide-bisacrylamide gel (Acrylamide: N,N′-metylene bisacrylamide 37,5:1, Fluka, Germany) with a denaturing agent gradient (urea) in the range from 30 to 60%. The band patterns in the gels were stained with fluorescent dye SybrGold (1:10,000, Invitrogen, USA), visualized with UV transillumination, captured and analyzed by the application of KODAK 1 D 3.6 Image Analysis Software (Eastman Kodak Company, USA).

#### Phylogenetic affiliation of the dominant denitrifying genera

To determine the phylogenetic affiliation of the dominant denitrifying bacteria after 53 days of biomass acclimation, an sludge sample was collected from the studied bench-scale bioreactor for genomic DNA extraction. Gene libraries of *nirS* and *nirK* genes were constructed by the PCR amplification with *cd3aF* / *R3cd* [28] and the *F1aCu*/*R3Cu* [29] primer pairs, respectively. Cloning of the individual amplicons was performed with InsTAclone™ PCR Cloning Kit (Fermentas, USA) in accordance with the manufacturer protocol. Positively verified clones were subjected to plasmid DNA extraction that was subsequently sequenced by Macrogen Europe (Amsterdam, Netherlands). The DNA sequences of the *nirS* and *nirK* gene fragments were validated by translation with EMBOSS Transeq tools included in EMBL-EBI service resources (http://www.ebi.ac.uk) [30]. Positively verified sequences were compared with the sequences of the cultivated microorganisms found in the Gene Bank (http://www.ncbi.nlm.nih.gov) to identify those that showed the highest degree of similarity and select marker sequences. MEGA 6.06 software package [31] was applied to align the maker, analyze DNA sequences with the ClustalW algorithm, and construct phylogenetic trees using the neighbor-joining method [32]. The obtained DNA sequences were deposited in the Gene Bank (NCBI) at accession numbers KP662361-KP662388 for *nirS* and KP662417-KP6640 for *nirK* D gene fragment DNA sequences. The detailed information about 16S rDNA PCR-DGGE and clone libraries protocols can be found in the Supporting Information (SI).

## Results and discussion

### Characteristics of the examined distillery byproducts

Fusel oil is a mixture of volatile organic acids, higher alcohols (especially isoamyl, isobutyl, active-amyl, butyl and propyl alcohol), aldehydes, ketones, fatty acids and esters. The amount of fusel oil varies between 0.1 and 1.1% relative to the produced ethanol during fermentation [33]. In the present study, the samples of fusel oil predominantly consisted of a few higher-chain alcohols and the dominating component was 2-methyl-1-butanol (on average 56% by weight) and the other important components, which contributed to approximately 42% by weight, comprised 2-methyl-1-propanol (18%), 3-methyl-1-butanol (11%) ethanol (10%) and n-propanol (3%). The remaining 25 examined compounds contributed to approximately 2% by weight. Results of the detailed analyses with the gas chromatography of 10 fusel oil samples from 7 different distilleries can be found in the SI (Figure SI-1, Table SI-1). For comparison, in the study of Mayer et al. (2015) [33], the composition of fusel oil was very similar and the specific contributions were as follows: C_5_H_12_O (63%), C_4_H_10_O (22%), CH_3_CH_2_OH (8%), and C_3_H_7_OH (3%).

### Effect of the examined EOCS on EBPR and denitrification

**Fig. 2:**
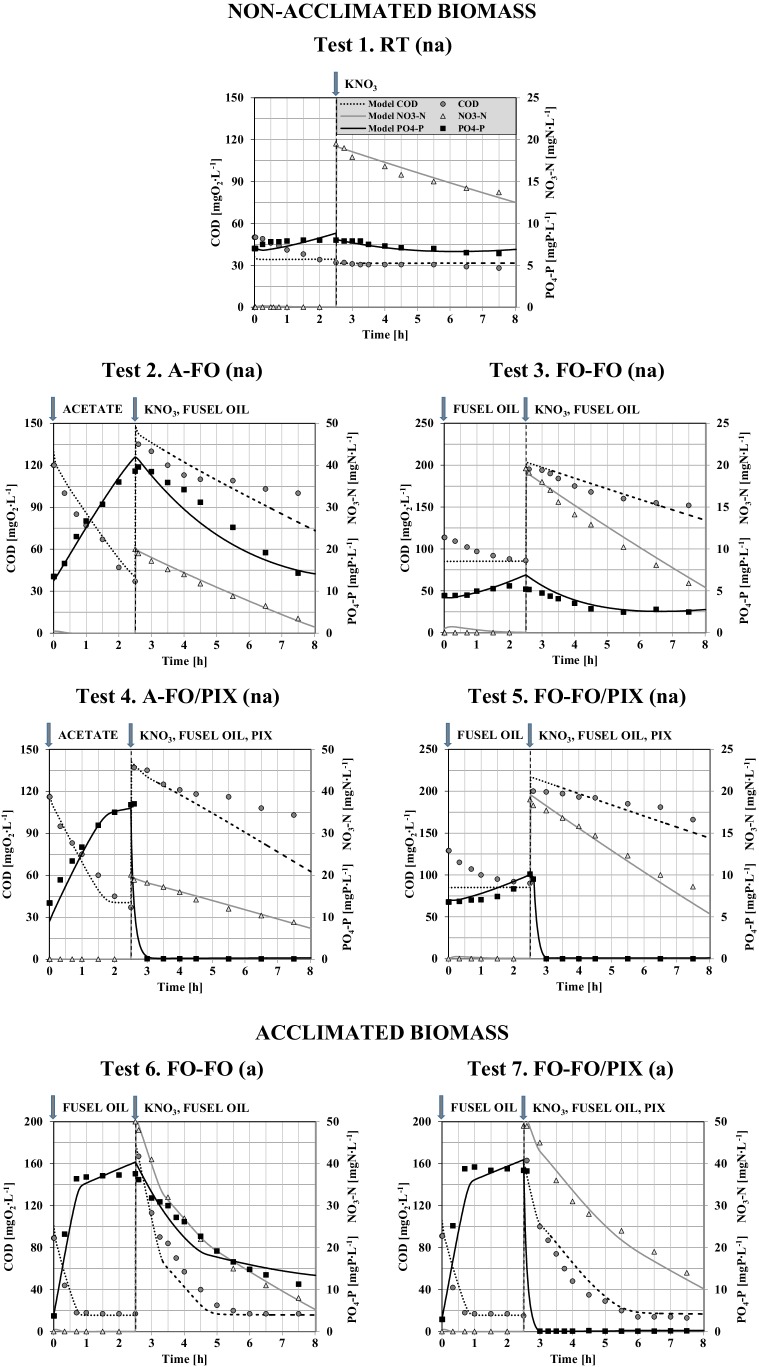
Behaviors of COD, PO_4_-P, and NO_3_-N in the two-phase experiments with non-acclimated and acclimated biomass in response to dosing different EOCS

**Fig. 3:**
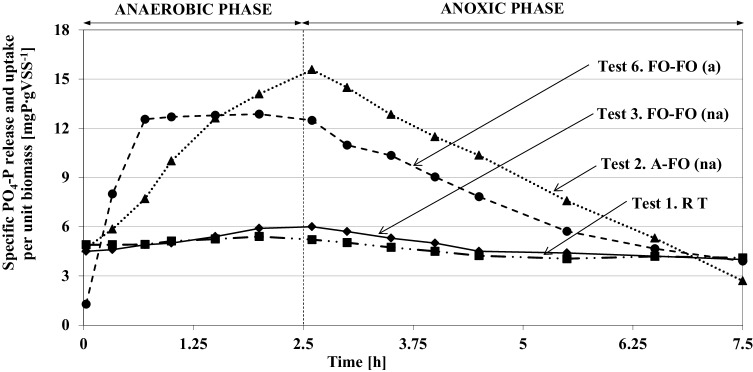
Observed behavior of PO_4_-P in the two-phase experiments with the non-acclimated biomass (Test 2 and 3) and acclimated biomass (Test 6) in response to dosing different EOCS (without chemical precipitation of PO_4_-P at the beginning of the anoxic phase)

The full PO_4_-P and NO_3_-N profiles observed during the anaerobic-anoxic experiments with the non-acclimated and acclimated biomass in response to dosing the different EOCS (in both anaerobic and anoxic phase) are shown in Fig. 2. The relevant calculations of the process rates and removal efficiencies are summarized in Table 4. A comparison of the PO_4_-P behaviors during the experiments with the non-acclimated and acclimated biomass is presented in Fig. 3.

**Table 4:** Summary of the PRR, PUR, and NUR (average values ± standard deviations, *n* = 3–4 repetitions for each experiment) and removal efficiencies for the non- and acclimated biomass in response to dosing different EOCS

Parameter	Non-acclimated biomass					Acclimated biomass	
	Test 1 RT (na)	Test 2 A-FO (na)	Test 3 FO-FO (na)	Test 4 A-FO/PIX (na)	Test 5 FO-FO/PIX (na)	Test 6 FO-FO (a)	Test 7 FO-FO/PIX (a)
EOCS dosed in the anaerobic phase	Not added	Acetate	Fusel oil	Acetate	Fusel oil	Fusel oil	Fusel oil
PRR, mgP.g_VSS_^−1^.h^−1^	0.40 ± 0.03	4.4 ± 0.4	0.70 ± 0.04	4.3 ± 0.6	0.60 ± 0.05	18.3 ± 1.1	17.9 ± 1.3
COD removal efficiency in the anaerobic phase, %	27.2 ± 2.5	69.4 ± 5.5	25.5 ± 3.1	68.1 ± 5.1	23.9 ± 1.7	85.1 ± 3.9	83.5 ± 1.6
ΔPrelease/ΔCODuptake	0.06 ± 0.01	0.27 ± 0.02	0.07 ± 0.01	0.29 ± 0.01	0.08 ± 0.01	0.47 ± 0.02	0.46 ± 0.02

EOCS dosed in the anoxic phase/chemical PO_4_-P precipitation	Not added	Fusel oil	Fusel oil	Fusel oil/PIX 113	Fusel oil/PIX 113	Fusel oil	Fusel oil
PUR, mgP.g_VSS_^−1^.h^−1^	0.50 ± 0.05	2.6 ± 0.1	0.60 ± 0.04	–	–	2.10 ± 0.06	–
NUR, mgN.g_VSS_^−1^.h^−1^	0.70 ± 0.09	1.30 ± 0.06	1.40 ± 0.07	1.00 ± 0.04	1.10 ± 0.09	3.7 ± 0.1	3.0 ± 0.1
PO_4_-P removal efficiency in the anoxic phase, %	12.3 ± 1.1	41.8 ± 1.9	15.8 ± 1.5	–	–	–	–
NO_3_-N removal efficiency in the anoxic phase, %	32.5 ± 1.2	82.3 ± 2.0	68.1 ± 1.1	56.0 ± 2.2	44.6 ± 1.5	86.4 ± 1.7	66.2 ± 1.3
COD removal efficiency in the anoxic phase, %	15.5 ± 3.1	25.8 ± 4.2	17.5 ± 1.8	22.2 ± 3.3	15.2 ± 2.1	84.9 ± 2.3	79.2 ± 5.3

#### Phosphate anaerobic release and anoxic uptake rates

The PRR was dependent on the specific EOCS added at the beginning of the anaerobic phase. In the tests with the non-acclimated biomass and acetate dosed (Tests 2 and 4), PO_4_-P was immediately released by the average COD consumption of 68.7 ± 2.5% (Table 4). The maximum PO_4_-P concentration and PRR reached 38.8 ± 2.6 mgP.L^−1^ (Fig. 2) and 4.4 ± 0.4 mgP.g_VSS_^−1^.h^−1^ (Table 4), respectively. Similar results were recently reported by Zhang et al. (2016) [34] who found that after addition of acetate in the anaerobic phase, the released PO_4_-P concentrations reached 25.0–34.0 mgP.L^−1^ by the COD consumption of 84.0 ± 2.0%. Furthermore, the observed PRRs were similar with results presented by Monclus et al. (2010) (2.1–5.0 mgP.g_VSS_^−1^.h^−1^) [35]. The present results revealed that PAOs in the non-acclimated biomass released up to 10.8 mgPO_4_–P.g^−1^VSS in response to adding acetate in the anaerobic phase (Fig. 3). These ratios were approximately 2.5 times higher than presented by Coats et al. [36], but 2–3 times lower than obtained by Onnis-Hayden et al. (2008) [37] for similar experiments with dosing of acetate. Wang et al. (2015) [38] showed in experiments with acetate that when the influent P concentration was 5.0 mgP.L^−1^, the amounts of anaerobic P release and anoxic P uptake were 4.7 and 5.8 mgP.g^−1^SS, respectively. On the contrary, when fusel oil was dosed in the present study (Test 3), the PO_4_-P profile followed the same low trend as in the reference test (Fig. 3). The addition of fusel oil had only a minor effect on PO_4_-P release and COD consumption in the anaerobic phase with the non-acclimated biomass (Test 3 and Test 5) (Fig. 2; Table 4). The average COD consumption was only 24.7 ± 2.5%. The average PRR = 0.65 ± 0.08 mgP.g_VSS_^−1^.h^−1^ was only slightly higher than the rate obtained in the reference test (0.40 ± 0.03 mgP.g_VSS_^−1^.h^−1^) (Table 4). Therefore, non-acclimated PAOs are not implicitly capable of using fusel oil, and the slight P release observed could also be related to the use of their intracellular storage compounds. For comparison, the PO_4_-P profile followed a similar trend as in the experiments with another distillery product (ethanol) as presented by Puig et al. [23] and Swinarski et al. [25]. Swinarski et al. [25] found that ethanol insignificantly induced the anaerobic P release. For comparison, Puig et al. [23] reported that the specific PRR in non-acclimated ethanol biomass from an bench-scale SBR reactor was close to the reference test (1.5 and 0.9 mgP.g_VSS_^−1^.h^−1^, respectively).

It should be noted that the behavior of PAOs under anoxic conditions was significantly different when adding acetate or fusel oil as the EOCS. When fusel oil was dosed in both phases (Test 3), the anoxic PUR and its removal efficiency was small (0.60 ± 0.04 mgP.g_VSS_^−1^.h^−1^ and 15.8 ± 1.5%, respectively) and similar to the rate observed in the reference test (Table 4). The low P-release and -uptake rates would confirm that fusel oil was not a suitable carbon source for EBPR in the non-acclimated biomass. On the contrary, the maximum PUR (2.6 ± 0.1 mgP.g_VSS_^−1^.h^−1^) by high PO_4_-P removal efficiency (41.8 ± 1.9%) was found in Test 2 after dosing acetate in the anaerobic phase. These observations confirmed the conclusion of Guerrero et al. (2011) [39] that the availability of VFA is the key factor in triggering the EBPR activity and the complex compound (e.g., distillery products) must be fermented to VFAs to maintain the EBPR activity.

An acclimation period of approximately 50 days was required to obtain maximum NURs in the bench-scale bioreactor [22]. During that period, the population of the activated sludge explicitly evolved also towards a more efficient use of fusel oil for the EBPR process as shown in the experiments with the acclimated biomass (Fig. 2). Similar results have been reported in studies with another distillery product [23]. These authors revealed that only after a period of biomass acclimation to ethanol (30–140 days), the population dynamics of the activated sludge evolved to an efficient phosphorus removal process. The present results revealed that PAOs in the acclimated biomass released up to 11.1 mgPO_4_-P.g^−1^VSS (Test 6) (Fig. 3). After the addition of fusel oil at the beginning of the anaerobic phase (Test 6 and Test 7), a rapid release of PO_4_-P was observed (average value = 39.1 ± 2.1 mgP.L^−1^) (Fig. 2). This ratio is much higher in comparison with the values from the corresponding experiments with the non-acclimated biomass. The maximum PRR was observed during the first 45 min when the readily biodegradable fraction of fusel oil was almost completely utilized (the average COD consumption = 84.3 ± 2.2%) and PUR was similar with experiments when acetate in the anaerobic phase was dosed (Test 2) (Table 4). In comparison with the results with the non-acclimated biomass, the PRRs and PURs increased from 0.70 ± 0.04 to 18.3 ± 1.1 mgP.g_VSS_^−1^.h^−1^ and from 0.60 ± 0.04 to 2.10 ± 0.06 mgP.g_VSS_^−1^.h^−1^, respectively.

#### Nitrate utilization rates

The maximum NUR was observed in the experiment with the addition fusel oil in the anoxic phase (followed the addition of acetate in the anaerobic phase). The rate reached 1.40 ± 0.07 mgN.g_VSS_^−1^.h^−1^ (Test 3) (Table 4) by the average net NO_3_-N removal − 16.4 ± 0.7 mgN.L^−1^ (Fig. 2). Furthermore, the NURs increased in comparison with the reference test by 0.60 ± 0.08 mgN.g_VSS_^−1^.h^−1^ (without chemical precipitation of PO_4_-P) (Test 2 and Test 3) and 0.30 ± 0.06 mgN.g_VSS_^−1^.h^−1^ (after chemical precipitation of PO_4_-P) (Test 4 and Test 5) (Table 4). Due to chemical precipitation of PO_4_-P, DPAOs reduced their metabolism, resulting in lower NURs which was necessary for respiration and intracellular transformation. Accordingly, it can be assumed that the difference between the N removal values before and after chemical precipitation of PO_4_-P could be related to the activity of DPAOs (storage of PO_4_-P at the expense of PHA), which was approximately 20% of the total NUR.

The results obtained with the acclimated biomass also revealed that NO_3_-N removal was significantly higher in comparison with the results obtained with the non-acclimated biomass. The observed NURs were apparently increasing from the initial value of less than 1.5 mgN.g_VSS_^−1^.h^−1^ [22] and reached the maximum of 3.7 ± 0.1 mgN.g_VSS_^−1^.h^−1^ on day 53 (Table 4). For comparison, similar acclimation periods (30–50 days) were reported for ethanol by other authors [23, 40]. When chemical precipitation of PO_4_-P was applied (Test 7), the NUR decreased to 3.0 ± 0.1 mgN.g_VSS_^−1^.h^−1^, respectively (Fig. 2; Table 4). Therefore, it can be assumed that the difference between the NURs before and after P precipitation was related to the activity of DPAOs (PO_4_-P uptake at the expense of stored PHA) and contributed to approximately 20% of the total NUR.

During the anoxic phase in Test 2 when acetate was dosed in the anaerobic phase, 1.28 mg PO_4_-P was consumed to reduce 1 mg NO_3_-N. These values remain in the range of the reported ratios of 0.60–1.31 mg.mg^−1^ in the EBPR processes with acetate or propionate as a sole EOCS [41–44]. In the present study, acclimated to fusel oil-biomass was characterized by a similar value, i.e., 1.11 mg PO_4_-P/1 mg NO_3_-N. For Test 3, with non-acclimated biomass when fusel oil was added in both phases, the consumption of PO_4_-P to reduce 1 mg NO_3_-N was similar with the result obtained in the reference test (0.34 mg PO_4_-P vs. 0.31 mg PO_4_-P). These results revealed that only after acclimation, the population dynamics of the activated sludge evolved to an efficient phosphorus and nitrogen removal process.

### Model-based evaluation of the contributions of DPAOs and DOHOs to the NURs

#### Simulations of the specific experiments

**Fig. 4:**
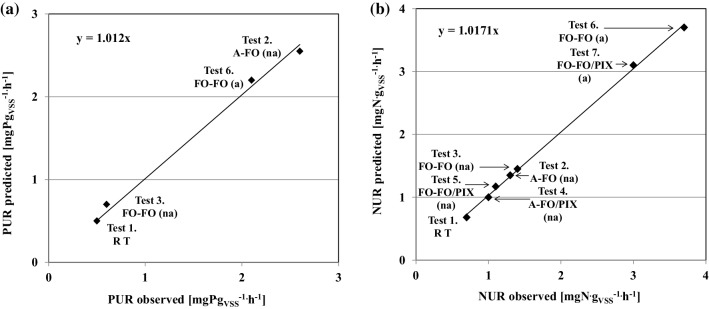
Comparison of the observed and predicted PURs (**a**) and NURs (**b**) during the anoxic phase of the two-phase batch experiments

**Fig. 5:**
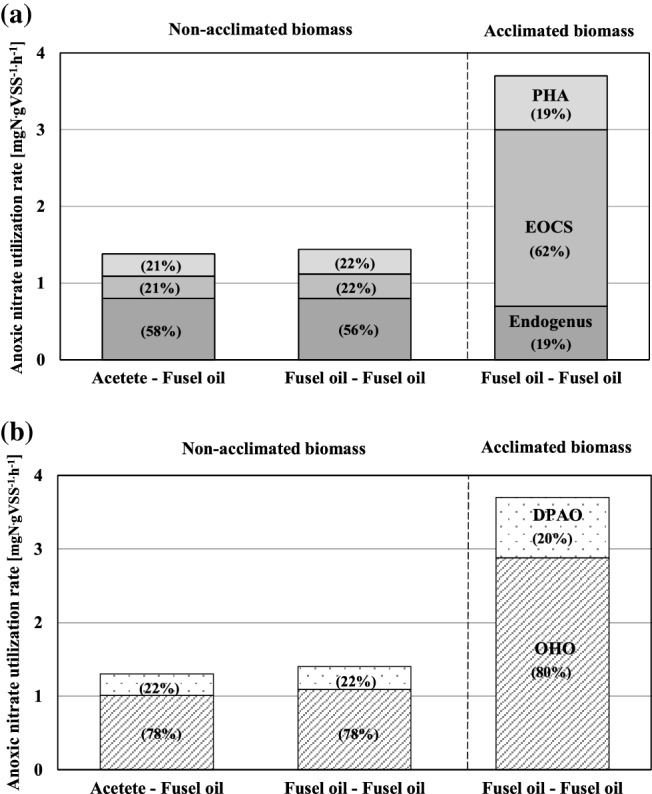
Estimated contributions of the specific carbon sources (**a**) and microbial activities (**b**) on to the NURs

Model simulations of the individual batch experiments and predicted contributions of DPAOs and DOHOs to the NURs are shown in Figure SI-2. Comparison of the observed and predicted PURs and NURs of the two-phase batch experiments are shown in Fig. 4. The observed NURs and PURs were very accurately predicted (*R*^2^ = 0.97–1.00). Estimated contributions of the specific carbon sources and microbial activities on to the NURs are shown in Fig. 5.

Only two kinetic parameters, including the rate constants for storage of PHA (*q*_PHA_) and polyphosphate (*q*_PP_), in the original set of model parameters [25] were modified to the values of 2.7 and 2.0 day^−1^, respectively, for the non-acclimated biomass. The effect of acclimation to fusel oil by PAOs was reflected by significant increases in the observed PRRs. Consequently, the value of q_PHA_ had to be increased to 7 day^−1^ to accurately predict that effect (Fig. 4).

The highest PAO contributions (> 30%) were predicted for the cases when acetate was added in the anaerobic phase (Figure SI-2). In contrast, the PAO contributions decreased below 20% in the experiments with the fusel oil added in the anaerobic phase (no stored PHA in that phase) as well as in the experiments with the acclimated biomass (better acclimation of OHO to fusel oil). For comparison, in the experiments with real wastewater, Hu et al. [45] and Makinia et al. [46] estimated that the NURs associated with the anoxic activities of DPAO (storage of phosphate and growth) constituted approximately 20% of the NURs associated with the anoxic activity of DOHOs.

#### Contributions of the specific carbon sources and microbial activities to NURs

In the non-acclimated biomass, approximately 60% and 20% of the total NUR was attributed to the utilization of endogenous carbon sources and examined EOCS, respectively (Fig. 5a). The remaining portion (approximately 20% of the total NUR) could be attributed to PHA utilization (linked to PO_4_-P uptake) by DPAOs. For the acclimated biomass, the contribution of the EOCS to the total NUR increased to approximately 60% and the contribution of the endogenous carbon sources decreased accordingly (Fig. 5a). The model simulations revealed that the acclimation rate was similar to both DPAO and DOHO (Fig. 5b). In both non-acclimated and acclimated biomass, the activity of DPAOs and DOHOs corresponded to approximately 20% and 80% of the total NUR, respectively.

### Microbial analysis

#### Microbial community structure stability during acclimation to fusel oil

Potential shifts in the structure of the microbial communities during acclimation to fusel oil were analyzed based on band patterns obtained using the 16S rRNA PCR-DGGE technique (Figure SI-3). In the inoculum sample, 14 unique bands were detected. During the cultivation, only one new band appeared in the sample from the 17th day of the operation. Due to the observed PUR increase in the system, the newly detected microorganism potentially plays a role of flanking species capable of transforming some fusel oil components to VFA. The remaining band patterns were stable during the entire cultivation period. This finding suggests that the applied operational conditions and fusel oil doses did not reflect selective pressure on activated sludge microorganisms. The increased NURs and PURs resulted from modulation of the bacterial cell physiological activity rather than quality shifts in the microbial community structure.

#### Phylogenetic analysis of denitrifying bacterial population based on nirS and nirK genes

The phylogenetic affiliation of denitrifying bacteria in the acclimated biomass from the studied bench-scale reactor with addition of fusel oil were analyzed by the clone libraries approach. As molecular markers, *nirS* and *nirK* genes encoding alternative forms of nitrite reductase were used. Results of the phylogenetic analysis are presented in Figure SI-4 and SI-5. As shown in Table 5, the analyzed PCR amplicons of *nirS* and *nir K* genes formed 28 OTUs (Operational Taxonomic Units > 98% sequence identity) grouped into 8 clades and 23 OTUs grouped in 5 clades, respectively. In the case of both genes, it was revealed that the main role in denitrification is played by members of *Alpha* and *Betaproteobacteria*. A subpopulation of the denitrifying bacteria which contained *nirS* gene was more diverse in comparison with the *nirK* gene holders. Potentially, bacteria which are able to synthesize cd 1-containing nitrite reductase *nirS* may acquire evolutionary advantage in wastewater treatment systems over bacteria harboring *nirK* gene. In terms of the *nirS* gene clone library, the most abundant clades belonged to *Azoarcus* genera and not-yet-classified members of the *Proteobacteria* phylum, each accounted for 22% of the clone library. Members of *Acidovorax sp., Alicycliphilus sp*., and *Thauera sp*. were also abundant. The clone library of *nirK* gene was outcompeted by the two clades of not-yet-specified bacteria genera; however, they grouped closely with the distinct members of *Rhisobiales* order. OTUs belonging to those clades constituted together more than 60% of the total *nirK* gene clone library. A significant content (23%) of denitrifiers harboring *nirK* gene were assigned to the *Nitrosomonas sp*. genus. Among the analyzed *nirS* and *nirK* gene variants, none reflected similarity to those specified for the “typical” PAO affiliated to *Accumulibacter sp*. Lv et al. (2014) [47] noted that *Dechloromonas sp.-*related microorganisms could potentially play a key role of DPAO in phosphorus removal systems in anaerobic/anoxic conditions. In the present study, their presence was confirmed by a single OTU in the clone library of *nirS* gene.

**Table 5:** Phylogenetic analysis of nirK and nirS genes

Target gene	Cluster	OTU numbers	Unique sequences numbers	Percentage of clone library (%)	Species affiliation
nirS	I	5	7	21.9%	Betaproteobacteria, Rhodocyclales, Zoogloeaceae, Azoarcus sp
	III	6	6	18.8%	Betaproteobacteria, Burkholderiales, Comamonadaceae, Acidovorax sp
	IV	1	1	3.1%	Betaproteobacteria, Burkholderiales, Burkholderiaceae, Cupriavidus sp
	V	1	1	3.1%	Alphaproteobacteria, Rhizobiales, Bradyrhizobiaceae, Bradyrhizobium sp
	VII	5	7	21.9%	Uncultured proteobacteria
	VIII	2	2	6.3%	Alphaproteobacteria, Rhodobacterales, Rhodobacteraceae, Paracoccus sp
	IX	4	4	12.5%	Betaproteobacteria, Burkholderiales, Comamonadaceae, Alicycliphilus sp
	X	1	1	3.1%	Betaproteobacteria, Rhodocyclales, Azonexaceae, Dechloromonas sp
		3	3	9.4%	Betaproteobacteria, Rhodocyclales, Zoogloeaceae, Thauera sp
	Total	28	32		
nirK	I	5	6	23.1%	Betaproteobacteria, Nitrosomonadales, Nitrosomonadaceae, Nitrosomonas sp
	III	8	8	30.8%	Uncultured proteobacteria
	IV	1	1	3.8%	Alphaproteobacteria, Rhizobiales, Rhizobiaceae, Rhizobium sp
	V	3	3	11.5%	Alphaproteobacteria, Rhizobiales, Phyllobacteriaceae, Mesorhizobium sp
	VI	6	8	30.8%	Uncultured alphaproteobacteria
	Total	23	26		

## Conclusions

Fusel oil was proved to be a viable EOCS for the combined nitrogen and phosphorus removal, but an acclimation of microbial community to fusel oil period (> 50 days) was required to effectively utilize that distillery product for denitrification and EBPR. In comparison with results with the non-acclimated biomass, the PRRs, PURs, and NURs increased from 0.70 ± 0.04 to 18.3 ± 1.1 mgP.g_VSS_^−1^.h^−1^, from 0.60 ± 0.04 to 2.10 ± 0.06 mgP.g_VSS_^−1^.h^−1^, and from 1.40 ± 0.07 to 3.7 ± 0.1 mgN.g_VSS_^−1^.h^−1^, respectively. In both non-acclimated and acclimated biomass, the activity of DPAOs corresponded to approximately 20% of the total NUR. The PCR-DGGE analysis showed a stable structure of microorganism consortia involved in the studied bench-scale bioreactor supported by the addition of fusel oil during acclimation stage. The occurrence of single novel band after 53 days of system operation along with the significantly increased PRRs, PURs, and NURs, reflects no selective pressure on the processed biomass in response to adding fusel oil, while the metabolic stimulation of heterotrophic bacteria associated with the fusel oil conversion to VFA was obtained. The denitrifying subpopulation was dominated by *Alpha* and *Betaproteobacteria* members. Furthermore, a greater diversity of *nirS* gene variants was observed which supports suppositions that bacteria capable of synthesizing nitrite reductase type S obtain the evolutionary advantage in wastewater treatment systems over *nirK* gene harboring microorganisms. The dominant DPAOs were implicitly *Dechloromonas* sp.-related bacteria.

## Electronic supplementary material

Below is the link to the electronic supplementary material.

Supplementary material 1 (DOCX 1265 KB)
