# Supplementary material for: The metabolic activity of denitrifying microorganisms accumulating polyphosphate in response to addition of fusel oil
Source: Bioprocess Biosyst Eng. 2018 Oct 5;42(1):143–55. doi: 10.1007/s00449-018-2022-0 (PMC6329743; doi:10.1007/s00449-018-2022-0)
Supplement: Supplementary file 1 — Supplementary material 1 (DOCX 1265 KB) [file 449_2018_2022_MOESM1_ESM.docx]

***Microbial community structure analysis – protocols supplementary data***

*16S rDNA PCR-DGGE*. The fragment of the 16S rRNA gene was amplified using F-968-GC and R-1401 primers proposed by Nübel et al. (1997). The PCR master mix consisted of the following components: 100 ng genomic DNA; 0.5 μM of each primer; 100 μM nucleotide mixture (Promega, U.S.A.); 0.6 U of Hypernova DNA polymerase (DNA-Gdansk, Poland); 3 μl 10x concentrated reaction buffer (100 mM Tris-HCl, 500 mM KCl, 1.5% Triton X-100); 1.5 mM MgCl_2_; the mixture was diluted with deionized water to a finale volume of 30 μl. PCR reactions were performed in the Gene-Amp® PCR System 9700 thermal cycler (Applied Biosystem, U.S.A.) with a temperature program consisting of: initial denaturation at 94 °C for 5 min; 35 cycles with denaturation at 94 °C for 45 s, primers annealing at 62 °C (which was lowered by 0.1 °C in subsequent cycles) for 45 s, elongation in 72 °C for 45 s, final elongation at 72 °C for 10 min. The PCR reaction for each trial carried out in triplicate. The qualitative analysis of products was made in 1% agarose gel, in the presence of 5 μg molecular marker (100 bp Ladder, Promega). After verification, the products from each replication were mixed together. During following steps PCR products were separated in a 6% acrylamide-bisacrylamide gel (Acrylamide: N,N’ – Metylene bisacrylamide 37,5:1, Fluka, Germany) with a denaturing agent gradient (urea) in a range from 30 to 60%. DGGE was carried out in the Dcode System electrophoresis apparatus (Bio-Rad Laboratories Inc., U.S.A.) in 1x concentrated TAE buffer (2M Tris, 40mM acetic acid, 1mM EDTA). The electrophoresis process lasted 12 hours, during which the temperature was kept constant at 60 °C and 60V voltage. The band patterns in the gels were visualized with fluorescent dye SybrGold (1:10 000, Invitrogen, U.S.A.) according to the manufacturer's recommendations. After rinsing off the excess dye, the gel was transilluminated UV radiation. The images of the band patterns were archived and analyzed using KODAK 1 D 3.6 Image Analysis Software (Eastman Kodak Company, U.S.A.).

*nirS and nirK clone libraries*. To construct gene libraries, fragments of the *nirS* and *nirK* genes were amplified using cd3aF / R3cd and the F1aCu / R3Cu primers pairs, respectively. Beside the primers, the same PCR mix components as in case of 16 S rRNA gene were applied. PCRs were performed in the Gene-Amp® PCR System 9700 thermocycler (Applied Biosystem, USA) with the following temperature profile: initial denaturation of 94 °C for 10 min; 32 cycles with denaturation at 94 °C for 60 s, primer assembly at 63 °C (which was lowered by 0.1 °C in subsequent cycles) for 60 s, elongation at 72 °C for 60 s, final elongation at 72 °C for 10 min. The qualitative analysis of the product was made in 1% agarose gel, in the presence of 5 μg molecular marker (100 bp Ladder, Promega). To clone individual amplicons InsTAclone™ PCR Cloning Kit (Fermentas, USA) was applied in accordance to manufacturer protocol. After selection of positive clones, plasmid DNA was extracted and sequenced by Macrogen Europe (Amsterdam, Netherlands). Obtained DNA sequences were aligned by appliance of ClustalW module constituted part of MEGA 6.06 software package. The sequences of the nirS and nirK gene fragments were checked by translating them with EMBOSS Transeq tools included in EMBL-EBI service resources (http://www.ebi.ac.uk). If codon stop was found and, when the amino acid composition significantly differed from the sequences derived from cultivated denitrification microorganisms, the given read was not included in further analysis. Positively verified sequences were compared with the sequences of cultivated microorganism found in the Gene Bank (NCBI, http://www.ncbi.nlm.nih.gov) to identify those that showed the highest degree of similarity and establish their taxonomic position. By the screening of the Gene Bank resources a set of marker sequences, derived from cultivated microorganisms, were selected. After alignment of the maker and analyzed sequences with the ClustalW algorithm a phylogenetic trees were constructed by the use of neighbor joining method. The topology of the obtained dendrograms were verified at bootstrap 500. The obtained DNA sequences have been deposited in the Gene Bank (NCBI) at accession numbers (KP662361 – KP662388 for *nirS* and KP662417 – KP6640 for *nirK* gene).

***Table SI-1.*** *The dominating component of fusel oil*

| Dominating component  of fusel oil | Molecular formula | Uses |
| --- | --- | --- |
| 2-methyl-1-butanol | C_5_H_12_O | It is used as a solvent and an intermediate in the manufacture of other chemicals. 2-Methyl-1-butanol is a component of many mixtures of amyl alcohols sold industrially. *(Human Metabolome Database)* |
| 2-methyl-1-propanol (isobutanol) | C_4_H_10_O | Isobutanol is widely used in industry, as a solvent in chemical reactions, as well as being a useful starting material for organic synthesis. Exposure to high concentrations of its vapour can cause temporary narcosis. Isobutanol is occasionally found as a volatile component of urine and arises from gut microbial metabolism. Isobutanol is used as one of the markers to measure occupational exposure to a mixture of solvents. Aliphatic alcohols levels increase in both diabetes mellitus and insulin-dependent diabetes patients. *(Human Metabolome Database)* |
| 3-methyl-1-butanol  (isopentanol or isoamyl) | C_5_H_12_O | Isopentanol or isoamyl alcohol is one of several isomers of amyl alcohol. It is a by-product of gut microbial fermentation. Isoamyl alcohol is the major higher chain alcohol in alcoholic beverages and is present in cider, mead, beer, wine, and spirits to varying degrees, being obtained by the fermentation of starches. Isopentanol has been shown to induce expression of CYP3A and CYP2E1 in human liver. *(Human Metabolome Database)* |
| ethanol | CH_3_CH_2_OH | Ethanol is used often as a topical disinfectant. It is widely used as a solvent and preservative in pharmaceutical preparations as well as serving as the primary ingredient in alcoholic beverages. Ethanol has widespread use as a solvent of substances intended for human contact or consumption, including scents, flavorings, colorings, and medicines. Industrially, ethanol is produced both as a petrochemical, through the hydration of ethylene, and biologically, by fermenting sugars with yeast. Small amounts of ethanol are endogenously produced by gut microflora through anaerobic fermentation. However most ethanol detected in biofluids and tissues likely comes from consumption of alcoholic beverages. Absolute ethanol or anhydrous alcohol generally refers to purified ethanol, containing no more than one percent water. *(Human Metabolome Database)* |
| n-propanol | C_3_H_7_OH | It is used as a solvent in the pharmaceutical industry, and for resins and cellulose esters. It is formed naturally in small amounts during many fermentation processes and small amounts are produced by gut microflora. *(Human Metabolome Database)* |

***Figure SI-1.*** *The concentration of 29 selected organic compounds in 10 samples of fusel oil from seven different distilleries (in ppm)*

| **NON-ACCLIMATED BIOMASS** | |
| --- | --- |
| **Test 1. RT (na)** | |
| 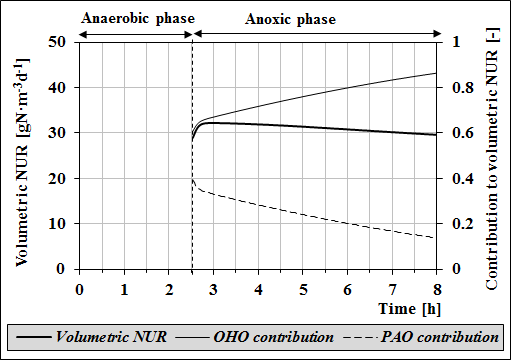 | |
| **Test 2. A-FO (na)** | **Test 3. FO-FO (na)** |
| 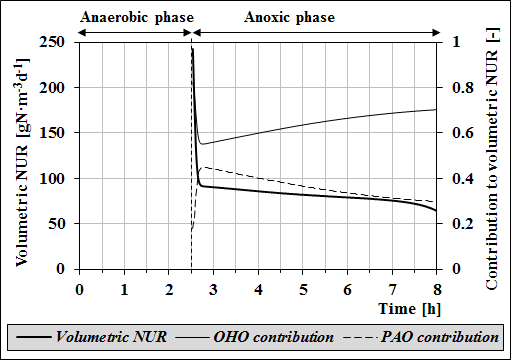 | 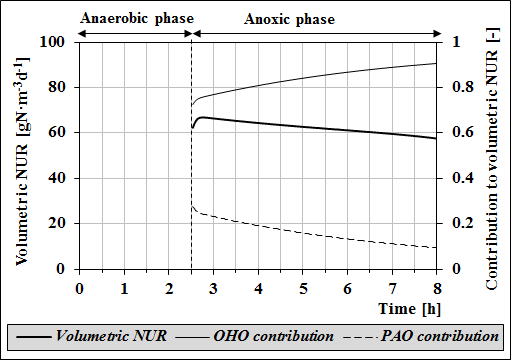 |
| **Test 4. A-FO/PIX (na)** | **Test 5. FO-FO/PIX (na)** |
| 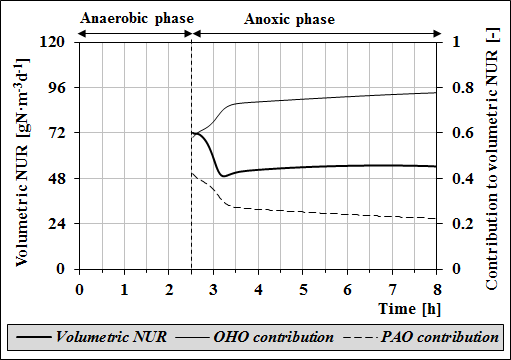 | 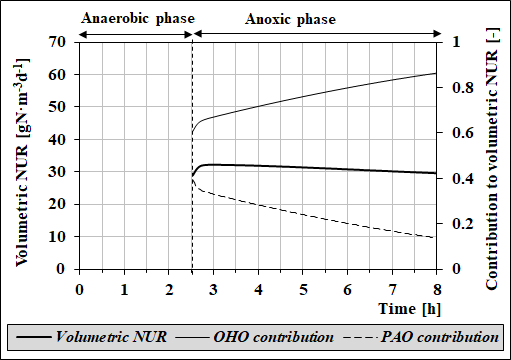 |
| **ACCLIMATED BIOMASS** | |
| **Test 6. FO-FO (a)** | **Test 7. FO-FO/PIX (a)** |
| 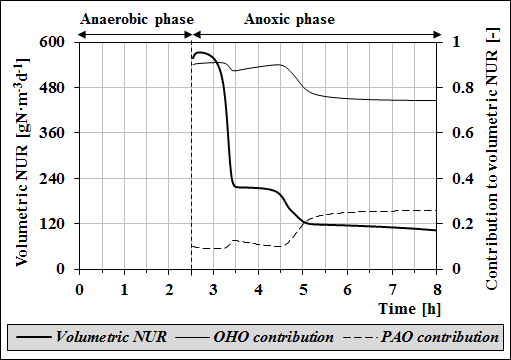 | 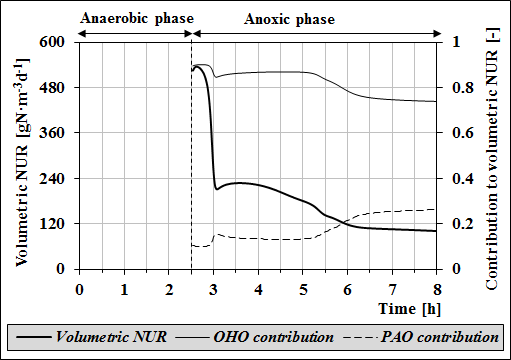 |

***Figure SI-2.*** *Predicted volumetric NURs and contributions of DOHOs and DPAOs to the NURs in the two-phase experiments with non-acclimated and acclimated biomass in response to dosing different EOCS*

***Figure SI-3.*** *DGGE profile of PCR amplified 16S rRNA gene fragments of samples collected from a bench scale reactor with fusel oil addition*


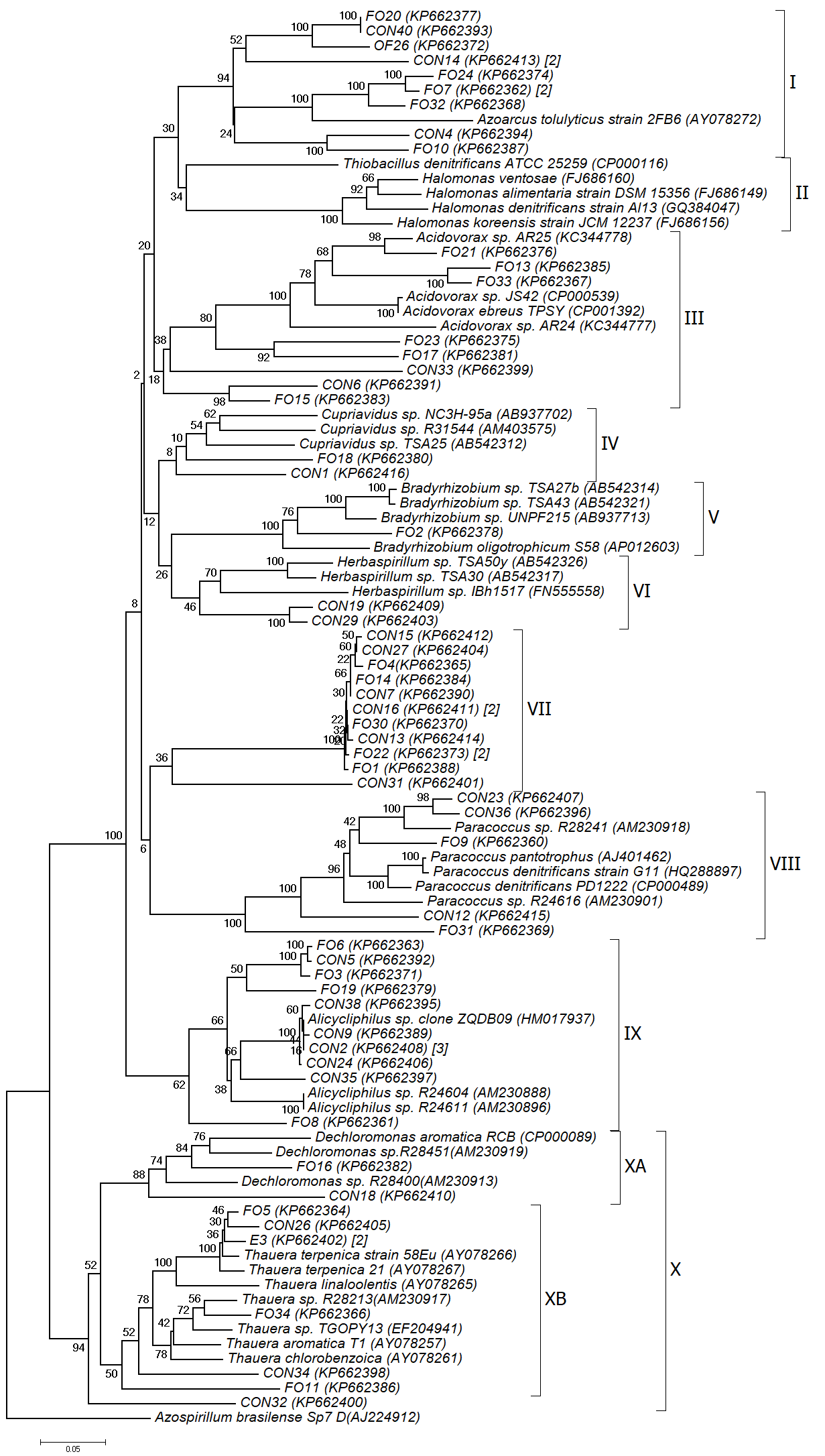


***Figure SI-4.*** *Phylogenetic analysis of nucleotide sequences of the nirS gene obtained from activated sludge samples from the bench scale reactor with fusel addition (FO samples) and scale reference reactor (CON samples). The rooted phylogenetic tree was built on the basis of 96 sequences (57 analyzed clones and 39 maker DNA sequences obtained from the Gene Bank), using the algorithm of combining neighboring taxa. The figure shows self-sampling values equal or above 30% for 500 repetitions. In parentheses ( ) the accession accession numbers from the Gene Bank are shown, in square brackets [ ] the number of repetitions of the same sequence within the analyzed gene library*


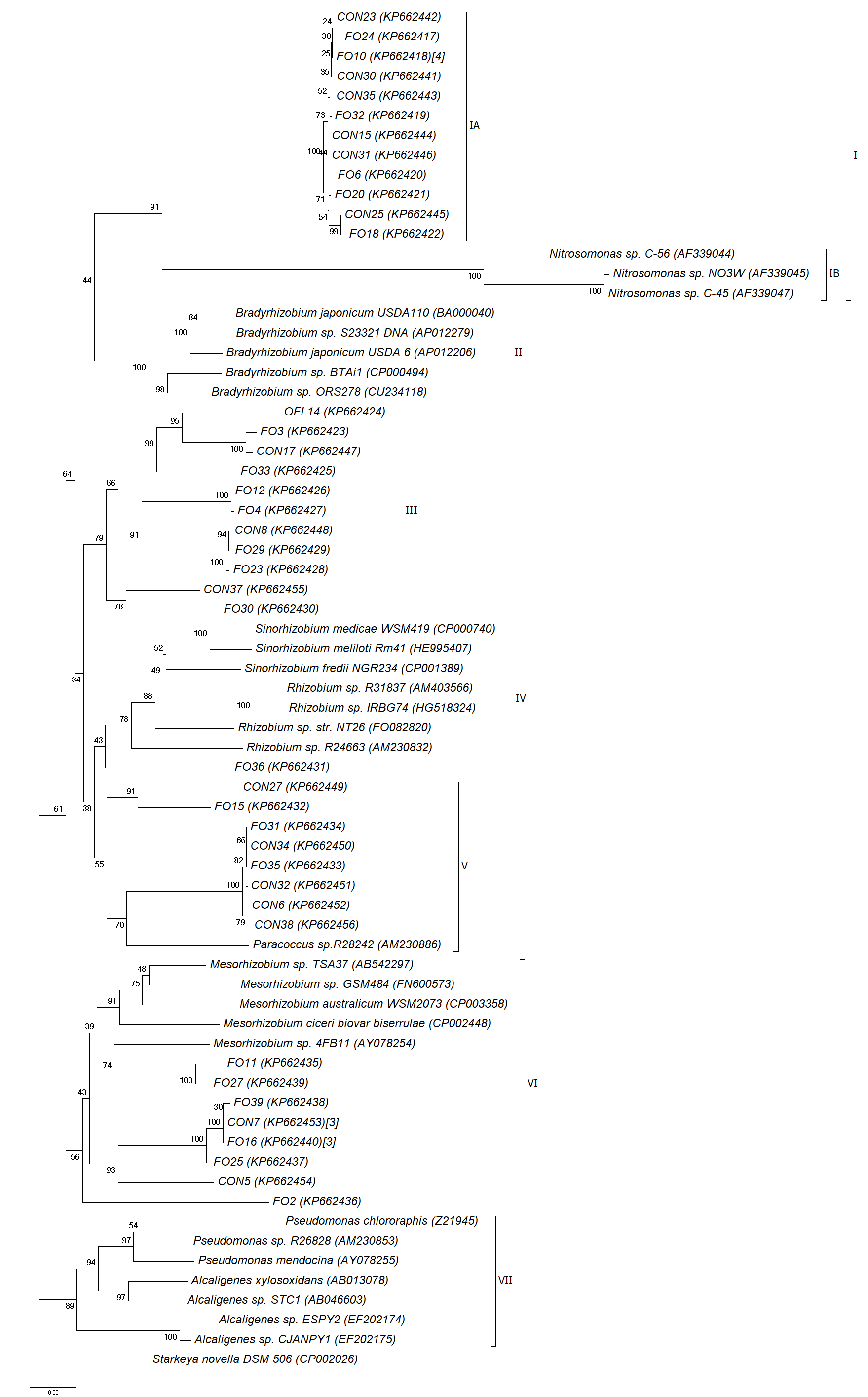


***Figure SI-5.*** *Phylogenetic analysis of the nirK gene sequence obtained from activated sludge samples from the bench scale reactor with fusel addition (FO samples) and reference reactor (CON samples). The rooted phylogenetic tree was built on the basis of 69 sequences (40 analyzed clones and 29 maker sequences obtained from the Gene Bank), using the algorithm of combining neighboring taxa. The figure shows self-sampling values equal or above 30% for 500 repetitions. In parentheses () the accession accession numbers from the Gene Bank are shown, in square brackets [] the number of repetitions of the same sequence within the analyzed library*
